# TRIM59 Is a Novel Marker of Poor Prognosis and Promotes Malignant Progression of Ovarian Cancer by Inducing Annexin A2 Expression : Erratum

**DOI:** 10.7150/ijbs.69329

**Published:** 2022-01-01

**Authors:** You Wang, Zhicheng Zhou, Xinran Wang, Xuping Zhang, Yansu Chen, Jin Bai, Wen Di

**Affiliations:** 1 Department of Obstetrics and Gynecology, Renji Hospital, School of Medicine, Shanghai Jiao Tong University, Shanghai 200001, China;; 2 Shanghai Key Laboratory of Gynecologic Oncology, Focus Construction Subject of Shanghai Education Department, Shanghai;; 3 Department of Molecular and Cellular Biology, Baylor College of Medicine, Houston, TX 77030, USA;; 4 Cancer Institute, Xuzhou Medical University, Xuzhou 221002, Jiangsu Province, China;; 5 Jiangsu Center for the Collaboration and Innovation of Cancer Biotherapy, Cancer Institute, Xuzhou Medical University, Xuzhou 221002, Jiangsu Province, China.

Following the publication of our article, the authors noted one error in Fig. 5C. The microscopic image for Scramble group on the left was misused. The authors carefully checked the original data and found that the image for Blank control was mistaken as the Scramble control. All the original pictures from this experiment (including those sphere pictures shown in the published manuscript) were taken on the same day (2012-July-27). Several years later, a different co-author prepared the figures and made this mistake before submission. The wrong image in Fig. 5C has been replaced with the right one. The authors confirm that the mistake does not affect the results or conclusions of the study and apologize for any inconvenience caused by this mistake. The corrected and old figures are provided below.

## Figures and Tables

**Figure 5:**
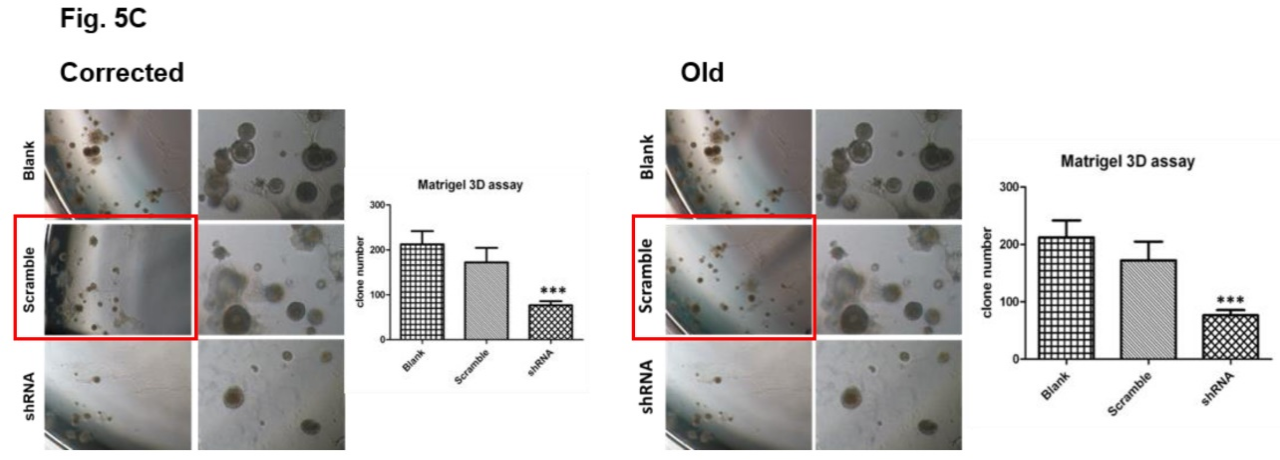
C. Correction.

